# The RSV F p27 peptide: current knowledge, important questions

**DOI:** 10.3389/fmicb.2023.1219846

**Published:** 2023-06-21

**Authors:** Wanderson Rezende, Hadley E. Neal, Rebecca E. Dutch, Pedro A. Piedra

**Affiliations:** ^1^Department of Molecular Virology and Microbiology, Baylor College of Medicine, Houston, TX, United States; ^2^Department of Pharmacology, Baylor College of Medicine, Houston, TX, United States; ^3^Department of Molecular and Cellular Biochemistry, University of Kentucky, Lexington, KY, United States; ^4^Department of Pediatrics, Baylor College of Medicine, Houston, TX, United States

**Keywords:** respiratory syncytial virus (RSV), p27, fusion protein (F), cleavage, F protein trimer

## Abstract

Respiratory syncytial virus (RSV) remains a leading cause of hospitalizations and death for young children and adults over 65. The worldwide impact of RSV has prioritized the search for an RSV vaccine, with most targeting the critical fusion (F) protein. However, questions remain about the mechanism of RSV entry and RSV F triggering and fusion promotion. This review highlights these questions, specifically those surrounding a cleaved 27 amino acids long peptide within F, p27.

## 1. The RSV F protein: cleavage sites and the p27 peptide

Respiratory syncytial virus (RSV) remains a leading cause of hospitalizations and death for young children and adults over 65 (Rha et al., 2020; McLaughlin et al., 2022). RSV is an enveloped, single-stranded, negative-sense RNA virus belonging to the *Pneumoviridae* family (Amarasinghe et al., 2019). Similar to paramyxoviruses, pneumoviruses consist of a nucleocapsid protein complex (a nucleocapsid protein (N) encapsidating the genetic material, the polymerase (L) and polymerase co-factor, P (phosphoprotein)), a matrix (M) protein layer linking the nucleocapsid protein complex with the phospholipid envelope, and three transmembrane glycoproteins (King et al., 2012). The fusion protein (F) and attachment (G) glycoproteins promote membrane fusion and viral entry. Functional and structural studies suggest that the pneumovirus small hydrophobic (SH) protein forms pH-dependent viroporin that regulates membrane permeability, infectivity, and prevent host cell apoptosis (Fuentes et al., 2007; Gan et al., 2012; Masante et al., 2014).

RSV F is synthesized as an inactive precursor (F0) which undergoes cleavage by host cell proteases to yield two disulfide-linked subunits, F1 and F2 (Collins and Mottett, 1991; Day et al., 2006), which are fusion competent. Fusion active F is in a metastable “prefusion” state until a triggering event induces conformational changes, exposing the fusion peptide, which inserts into the target membrane, followed by formation of a six-helix bundle which is hypothesized to drive membrane fusion (Smith et al., 2009; King et al., 2012). This process is similar for all paramyxo- and pneumoviruses; however, RSV F has several differences that remain to be fully understood.

**Figure 1:**
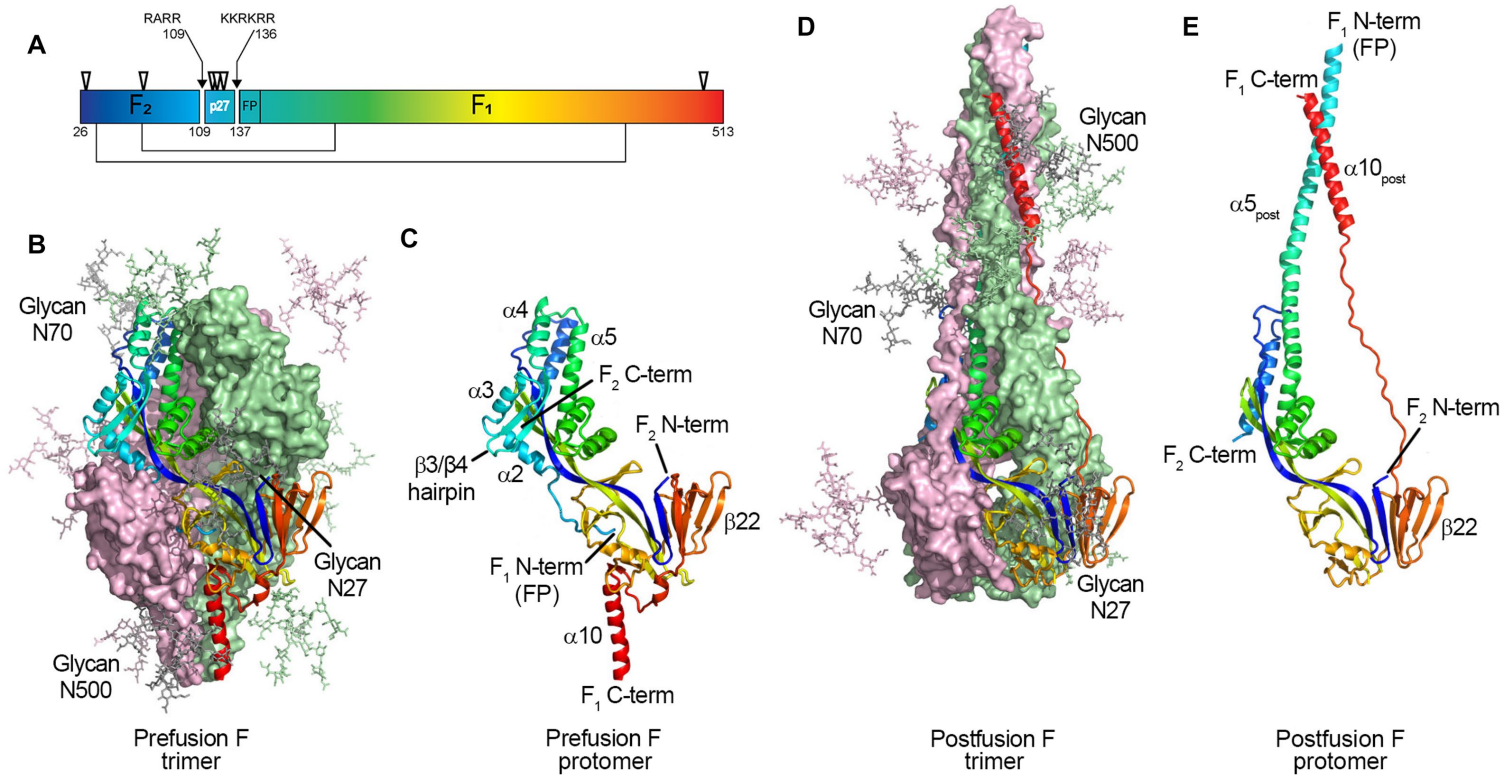
Structure of the RSV F protein on prefusion and postfusion conformations. **(A)** primary structure showing the disulfide bonds between F1 and F2 subunits (thin lines), N-glycosylation sites (▽), and the Fusion Peptide (FP) on F1 N-term; the p27 peptide is shown between cleavage sites R109 and R136 (arrows). F protein trimer on the **(B)** prefusion and **(D)** postfusion conformations with N-Glycans N27, N70, and N500 modeled as sticks. F1 + F2 protomers on prefusion **(C)** and postfusion **(E)** conformations. The RSV F protein prefusion trimer **(B)** is formed by the interaction between three F1 + F2 protomers **(C)**. In the process of viral entry, the prefusion trimer **(B)** undergoes structural rearrangement to a final postfusion conformation **(D)**. The FP (F_1_ N-term), β3/β4 hairpin, and alpha-helices α2, α3, and α4 rearrange, fusing with α5 **(C)** to form an extended postfusion helix, α5_post_ **(E)**; the prefusion β22 parallel strand unravels so the α10 helix **(C)** can meet α5_post_, finalizing the postfusion conformation **(E)**. Although not shown in crystal structures, the p27 peptide remains at the F1 N-term when partially cleaved, capping the Fusion Peptide, which hinders the fusion efficiency. From McLellan et al. (2013b). Structure of RSV Fusion Glycoprotein Trimer Bound to a Prefusion-Specific Neutralizing Antibody. Science (80- ) 340:1113–1117. Reprinted with permission from AAAS.

Collins et al. (1984) and Elango et al. (1985), respectively, first sequenced RSV F, showing it is 574 amino acids with a polybasic motif (KKRKRR136) corresponding to a furin consensus site. Unlike closely related paramyxovirus F proteins, the polybasic sequence is six amino acids long, leading Bolt et al. to suggest that other proteases could be involved in cleavage (such as proprotein convertase 5 and 7; Basak et al., 2001) and activate RSV F (Bolt et al., 2000). However, it was not until 2001 that González-Reyes et al. (2001) and Zimmer et al. (2001a) independently demonstrated that RSV F is cleaved at two polybasic sites (RARR109 and KKRKRR136), generating two major subunits: the F2 subunit (20 kDa, AA 26 to 109), and F1 (50 kDa, AA 137 to 574), and releasing an internal peptide of 27 amino acids, termed p27 (AA 110–136) (Figure 1A). The fate of this fragment post-cleavage is still unknown.

Through site-directed mutagenesis of Bovine Respiratory Syncytial Virus (BRSV), Zimmer et al. (2002) determined that furin cleavage at site R109 impaired but did not abolish fusion activity *in vitro*, highlighting the importance of cleavage at site R136, which exposes the fusion peptide at the N-terminus of the F1 subunit. Rawling et al. (2011) generated chimeric mutations of Sendai virus (SeV) fusion to include one or both RSV furin recognition sites rather than the single cleavage site normally in SeV F. SeV F normally requires the HN attachment protein for fusion triggering, while. RSV F can promote fusion in cell culture without the G protein (Techaarpornkul et al., 2001). Interestingly, all SeV F/RSV cleavage site chimeric mutants formed syncytia without HN protein, suggesting that the ability of RSV F to fuse without the attachment protein is facilitated, at least in part, by the additional cleavage site.

There are two known RSV subtypes, A and B, classified as such by divergences in antigenic profile from the RSV/A Long strain (prototypic strain historically used for *in vitro* studies and vaccine development; McLellan et al., 2013c; Pandya et al., 2019). Hause et al. (2017) compared sequence variability of more than 1,000 RSV sequences of subtypes A and B to the RSV/A Long, showing that although the F protein is well-conserved across RSV genotypes, the p27 region of RSV/B strains exhibited significantly more non-synonymous amino acid changes than the RSV/A strains. However, entropy analysis – the measure of variability at each amino acid position – revealed that within the same subtype, several amino acid positions within the p27 sequence of RSV/As are more variable than in RSV/Bs.

As reported by Rajan et al. (2022), RSV infection in HEp-2 or A549 cells (commonly used for *in vitro* studies) have different viral growth kinetics and host response when infected with RSV/A or B. Additionally, the efficiency of p27 cleavage shows to be cell line dependent, as higher levels of mature F proteins retaining p27 are found on the surface of RSV-infected HEp-2 cells compared to A549 cells, independent of RSV subtypes (Rezende et al., 2023). On the other hand, cleavage of p27 is also subtype dependent, since F proteins from RSV/A were less efficiently cleaved (retaining more p27) than the F proteins expressed on the surface of cells infected with RSV/B (Rezende et al., 2023). Moreover, the authors showed that for both subtypes, the p27 cleavage efficiency declines over time (Rezende et al., 2023).

These studies highlight that despite a highly conserved F protein sequence among RSV subtypes and genotypes, the cleavage of p27 relies on host factors (e.g., enzyme turnover, vesicular transport, post-translational modifications, and innate immunity) rather than exclusively on enzymatic accessibility to cleavage sites.

## 2. Insights on the p27 glycosylation sites

Glycosylation is a crucial post-translational modification, as it impacts structure, function, stability, and translocation to the cell surface (Beyene et al., 2004; Vigerust and Shepherd, 2007; Ellgaard et al., 2016). RSV F has five N-linked glycosyolation sites which are well conserved among subtypes (N27, N70, N116, N126, and N500) (Zimmer et al., 2001b); with an additional N120 glycosylation site for some strains (Tan et al., 2012; Kimura et al., 2017). Two and for some strains three glycosylation sites are located within the p27 segment (N116, N120, and N126; Figure 1A).

Zimmer et al. (2001b) and Leemans et al. (2018) used systematic N – Q mutations to show that glycosylation in the p27 segment does not impact cleavage or transport of the F protein to the cell surface. Furthermore, F proteins containing the mutations N116Q or N126Q did not display molecular weight differences compared to wild-type. Therefore, Leemans et al. concluded that p27 was cleaved entirely off in a mature F protein. Both groups observed formation of larger syncytia in BSR T7/5 cells transfected with the mutant N116Q. Viral proteins can use glycosylation to shield antigenic sites, evading antibody recognition (Klink et al., 2006); however, Leemans et al. demonstrated that glycosylation of p27 at N116 and N126 did not significantly compromise binding of Palivizumab or other neutralizing antibodies targeting the prefusion conformation.

Leemans et al. (2019), incorporated the same mutations into recombinant viruses. During infection of HEp-2 cells, the molecular weight of the F proteins expressed by viruses encoding mutations N116Q or N126Q was comparable to F from wild-type virus. However, infection with mutant virus RSV F N116Q showed a decrease in syncytium formation compared to wild-type RSV. Although the presence of glycosylated p27 was not confirmed, changes in syncytium format *in vitro* and *in vivo* indicate that glycosylation of at least one site on p27 might have an important role in RSV biology.

## 3. The impact of p27 on RSV entry

**Figure 2:**
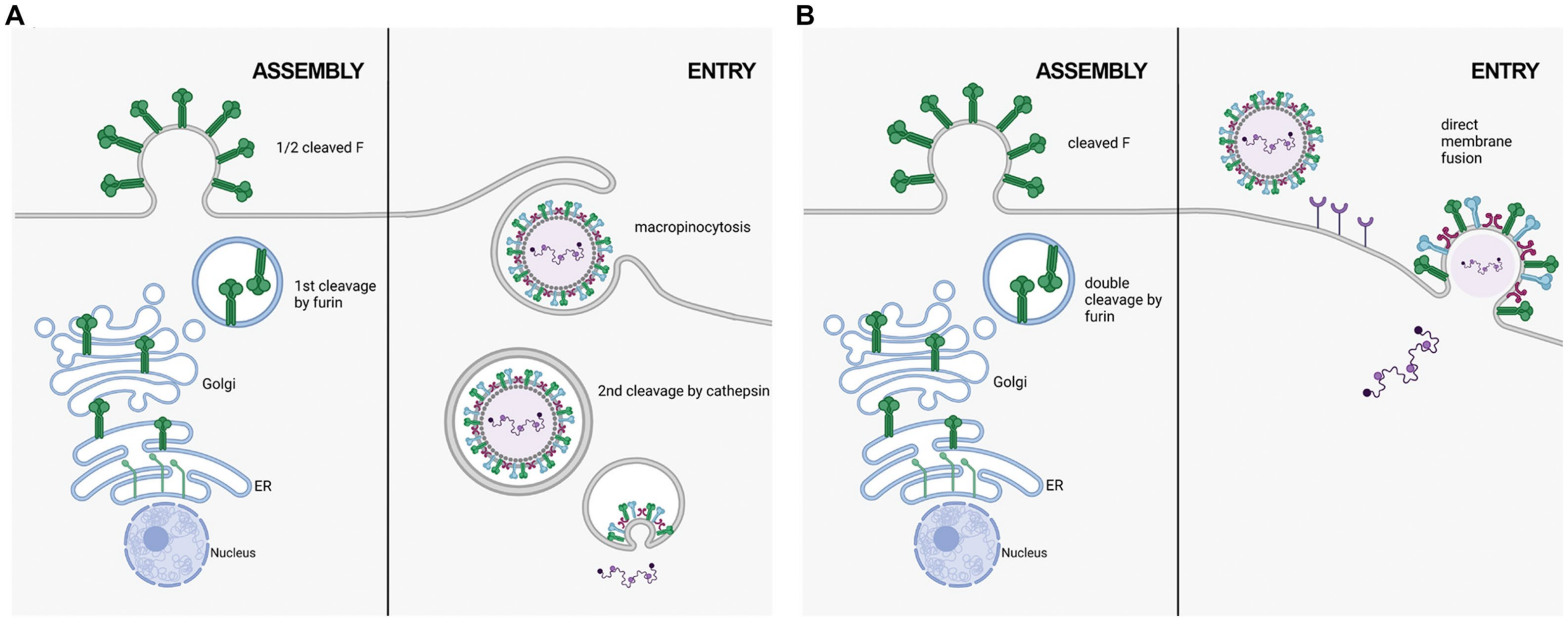
Two proposed mechanisms of RSV entry. **(A)** in the macropinocytosis method of entry, F is only cleaved at FCS1 in the trans golgi, expressing on the viral surface in a half-cleaved state. Newly synthesized virus enters host cells through macropinocytisis, allowing for FCS2 to be cleaved by cathepsin L in an endosome. This activates F, facilitating fusion with the endosomal membrane and releasing the viral genetic material into the host cell cytoplasm. **(B)** in the direct plasma membrane method of entry, F traffics through the secretory pathway to be cleaved at both FCS1 and FCS2 by furin, expressing on the viral surface in a fully cleaved state. Entry occurs through receptor binding, resulting in the fusion of the viral and host cell membranes facilitated by F.

RSV F on the cell surface is generally thought to be cleaved and fusogenically active (González-Reyes et al., 2001; Zimmer et al., 2001a). F0 could not be detected on the cell surface in an RSV infection model (Bolt et al., 2000). However, p27 was recently reported on the cell surface of infected cells, leading to the suggestion that uncleaved or partially cleaved F was on the cell surface (Lee et al., 2022). Krzyzaniak et al. (2013) also suggested that RSV F exists on the viral surface in a partially cleaved state (Figure 2). They detected peptides corresponding to the p27 region on purified RSV/A particles through Liquid Chromatography coupled with Mass Spectrometry (LC/MS) analysis. Western blot analysis of infected HeLa cells was consistent with cleavage at site R109 occurring before viral assembly, while cleavage at R136 occurred only after viral micropinocytosis upon viral entry (Krzyzaniak et al., 2013). However, contrary to the closely related human metapneumovirus (HMPV), RSV fusion is pH-independent, indicating that acidification of endosomes may not be required for cleavage of F, and consequently, for RSV entry (Srinivasakumar et al., 1991). The question of when both cleavage events occur has remained controversial, and additional work is needed to clarify the differing studies.

## 4. Insights on the impact of p27 on the F protein trimer

Fusion-competent RSV F is formed by non-covalent interactions between three disulfide-linked F1 and F2 protomers (Figures 1B–E) (McLellan et al., 2013c; Gilman et al., 2015; Krarup et al., 2015). Gilman et al. in 2015 characterized an RSV-neutralizing antibody, AM14, that recognizes cleaved, trimeric prefusion F (Gilman et al., 2015). AM14 binding was dependent on furin cleavage, either because of interference of p27 on AM14 binding through steric inhibition, or because F is unable to trimerize prior to cleavage. In the same year, a study by Krarup et al. determined that p27 destabilizes the protein trimer (Krarup et al., 2015). When incubating soluble F proteins in 0.1% SDS at room temperature, 50% of trimers without p27 were monomerized, while 97% of trimers containing p27 did (Krarup et al., 2015).

Gilman et al. evaluated stability of trimerized F using an antibody specific for soluble, prefusion F trimers (Gilman et al., 2019). The trimers of F1 + F2 heterodimers on the cell surface existed in dynamic equilibrium of associated-dissociated trimers, suggesting a “breathing” mechanism for the trimerization (Liu et al., 2008; Munro et al., 2014; Rutten et al., 2018).

## 5. The impact of p27 on F protein structure

The first evidence study of RSV F quaternary structure was in 2000 when Calder et al. used electron microscopy to show that F protein trimers from the RSV/A Long strain aggregated in rosette structures that were cone-like or lollipop-like rods (Calder et al., 2000). Morphological comparison between the RSV F protein and the parainfluenza 3 and 5 (PIV3 and PIV5) F protein structures indicated that cone-shaped trimers likely corresponded to a prefusion F (pre-triggered) while the lollipop-shaped trimers corresponded to a postfusion F (post-triggered) protein (Yin et al., 2005, 2006; Liljeroos et al., 2013).

Gozáles-Reyes et al. and Ruiz-Argüello et al. reported that complete enzymatic cleavage of F0 (release of p27) or partial cleavage at R136 alone (p27 remaining uncleaved from F2) led to the formation of rosettes and changes in morphology from cone to lollipop structures (González-Reyes et al., 2001; Ruiz-Argüello et al., 2002). However, Chaiwatpongsakorn et al., expressing F protein trimers from the RSV/A D53 strain, found cleavage of p27 was not the driving factor for morphological changes, but instead thermodynamic and physicochemical factors (e.g., temperature and low molarity) were the triggers (Chaiwatpongsakorn et al., 2011).

In 2011, McLellan et al. and Swanson et al. independently determined the crystal structure of the postfusion conformation of the F protein of RSV/A A2 strain. Both constructs truncated the initial portion of the fusion peptide to minimize aggregation, keeping the furin cleavage sites intact (McLellan et al., 2011; Swanson et al., 2011). Similar to parainfluenza viruses, the stalk portion of the postfusion F monomer is composed of two anti-parallel helices formed by the N- and C-terminus of the F1 subunit, juxtaposing the fusion peptide and transmembrane region. The stalk of the lollipop-shaped postfusion F is formed by a bundle of six alpha-helices, creating a thermodynamically stable structure. The absence of p27 on the postfusion structure was attributed to the complete cleavage of both furin sites during protein synthesis (Figure 1).

In 2012, Smith et al. reported the expression and purification of a near full-length F protein based on the RSV/A A2 strain (Smith et al., 2012), optimized by mutating the furin cleavage R136 (from KKRKRR136 to KKQKQQ136, cleaving F0 on R109 only) and deleting the first ten amino acids of the N-terminus of the F1 subunit. This construct generated antibodies targeting antigenic sites specific to pre- and postfusion RSV structures, including antibodies sharing the same antigenic site as Palivizumab.

McLellan et al. first published a partial structure of RSV F in the prefusion conformation in 2013 by co-crystalizing the prefusion-specific D25 antibody with a near wild-type F protein from the RSV/A A2 strain. The same group then published the structure of a prefusion construct without co-crystallizing monoclonal antibodies, named DS-Cav1 (McLellan et al., 2013a,b). The prefusion monomer structure is compact, made of two lobes (one proximal and one distal from the viral membrane) connected by two parallel beta-strands (one from F1 and one from F2) stabilized by several inter-monomer contacts. The membrane-proximal lobe from the neighboring monomer stabilizes the highly hydrophobic fusion peptide at the N-terminus of the F1 subunit. In 2015, a mutational analysis by Krarup et al. led to a model where p27 cleavage is needed to allow trimerization and fusion peptide burial in a hydrophobic cavity (Krarup et al., 2015).

The conformational change of RSV F trimers from prefusion to postfusion requires drastic rearrangements, which led Gilman et al. to study the dynamics of F in solution and on lipid membranes (Gilman et al., 2019). They observed that the prefusion trimer alternates between discrete open-and-closed states in a breathing-like motion similar to the HIV Env protein, and the same dynamics are observed on the surface of cells expressing the full-length RSV F protein. Moreover, on the surface of immortalized cell lines transfected with wild-type RSV F protein or F variants harboring a Foldon trimerization motif, the authors concluded that the F protein naturally exists in an equilibrium between monomer and trimer on cellular membranes.

To date, no structural determination method could characterize the most flexible regions of the F protein (the transmembrane domain, the cytoplasmic segment, and the p27 peptide region; Krueger et al., 2021). However, in 2021, Krueger et al., using small-angle neutron and small-angle X-ray scattering techniques (SANS and SAXS, respectively), determined the quaternary structure of prefusogenic F formulated on Polysorbate 80 nanoparticles and modeled the positioning of such regions within the trimeric structure (Krueger et al., 2021). When formulated on nanoparticles, trimeric prefusogenic F was recognized by monoclonal antibodies specific to either pre- or postfusion arrangements. Most importantly, prefusogenic F retains a partially cleaved p27, indicating that the flexibility of p27 did not destabilize the F protein trimeric arrangement. Moreover, RSV-infected cells displaying F protein trimers with partially cleaved protomers shows higher levels of surface F protein on prefusion conformation (Rezende et al., 2023); F protein trimers with detectable p27 are more thermally stable than those comprised of completely cleaved F proteins protomers (Rezende et al., 2023), although more studies are needed to evaluate the impact of p27 on RSV infectivity and replication cycle.

According to Krueger et al., in the prefusogenic structure, the p27 on the N-terminus of the F1 subunit would be solvent exposed, supporting Krarup’s observation that fusion peptide+p27 would not fit in the cavity formed within the prefusion structure (Krarup et al., 2015). On the other hand, the partially cleaved RSV F proteins can form trimers without the assistance of a Foldon trimerization motif (Krueger et al., 2021).

## 6. Humoral and mucosal immune response to p27 during natural infection

In 2016, Fuentes et al. published the first report demonstrating an immune response to p27 upon natural RSV infection (Fuentes et al., 2016). Looking at sera from five infants before and after their first RSV infection, the authors identified new antigenic sites using whole-genome-phage display libraries (GFPDL) encoding peptides covering the length of F. One new antigenic site mapped to p27. The authors then synthesized peptides covering the p27 region and surveyed serum samples from a cohort of children (<2 years old), adolescents (10–14 years old), and adults (30–45 years old) using Surface Plasmon Resonance (SPR). Although p27-binding antibodies were identified in all age groups, reactivity was higher in children than adolescents and lowest in adults. The authors suggested the immune response to p27 came from an uncleaved F0 precursor, present in immature virions and dying infected cells. Based on the work from Tapia et al., the immune pressure caused by the high mutation rate in the p27 region may be the driver for a high antibody response to p27 (Tapia et al., 2014).

Humoral and mucosal immunity to p27 was found in RSV infected hematopoietic cell transplant (HCT) recipients (Fuentes et al., 2019). Fuentes et al. again used GFPDL and SPR to examine blood serum and nasal washes of 11 HCT patients infected with RSV/A who stopped shedding the virus in less than 14 days (early recovery) or over 14 days (late recovery). Both groups developed antibodies recognizing p27. However, early-recovered patients generated mucosal antibodies with higher binding to p27 than late-recovered patients.

Leemans et al. evaluated the immune response to recombinant F proteins lacking glycosylation on N116 or N126. BALB/c mice immunized with plasmids encoding F N116Q generated an enhanced neutralizing antibody response compared to the control (Leemans et al., 2018). Immunization of BALB/c mice with a recombinant infectious RSV harboring the N116Q mutation (Leemans et al., 2019) generated higher neutralizing antibody titers compared to the wild-type RSV F virus.

Ye et al. quantified the amount of IgG, IgA, and p27-like antibodies (P27LA, natural antibodies capable of competing with a monoclonal anti-p27) in serum and nasal washes of 33 HCT patients during (acute) and post (convalescent) RSV infection (Ye et al., 2020). Anti-p27 IgG and IgA concentrations in both serum and nasal wash samples were about 1,000-fold lower than other F-specific sites (Ye et al., 2018, 2019). P27LA also showed a 1,000-fold lower concentration level than the correlate of immunity PCA (Palivizumab Competitive Antibody; Ye et al., 2018, 2019). Anti-p27 antibodies did not appear to improve the overall neutralizing antibody activity against RSV. However, a reduction in antibody concentration in nasal wash samples from convalescent HCT patients suggests that mucosal anti-p27 antibodies bind to either released viruses or virus-infected epithelial cells, aiding in controlling respiratory tract infection.

In 2019, Patel et al. demonstrated that prefusogenic F protein formulated in nanoparticles is recognized by pre-F specific monoclonal antibodies (antigenic sites Ø and VIII – also called V) and by monoclonal antibodies targeting antigenic sites shared between pre-F and post-F conformations (sites II and IV; Patel et al., 2019). The prefusogenic F protein also elicited significant levels of functional neutralizing antibodies and competitive antibodies to antigenic sites Ø, VIII, II, IV, and p27 in a challenge cotton rat model, preventing viral replication in the lungs with no significant histopathology. The prefusogenic nanoparticle formulation was further developed into a vaccine candidate for maternal immunization, eliciting a strong, broad antibody response and neutralizing antibody activity. It was the first RSV vaccine candidate targeting protection of newborns by vaccinating pregnant individuals during late gestation showing efficient antibody transplacental transfer [reviewed elsewhere (Blunck et al., 2021)].

Blunck et al. (2022) reported the kinetics of immunity to p27 in healthy adults under age 65, naturally infected with RSV/A or RSV/B during the 2018–2019 RSV season. The cohort of 19 subjects was divided into uninfected, acutely infected, and recently infected individuals based on levels of neutralizing antibody titers. As observed in HCT patients, all subjects presented detectable anti-p27 IgG and IgA levels. Throughout the study, uninfected individuals maintained constant levels of serum IgG anti-p27, while acutely infected and recently infected individuals experienced an increase and decrease in anti-p27 antibodies, respectively. However, p27 was not an immunodominant epitope in this cohort of healthy adults.

## 7. p27 *in vitro* and *in vivo*: first evidence of p27 detection on the surface of infected cells and histopathology sections

To better understand the protective antigenic sites within F, Lee et al. (2022) chemically synthesized peptides spanning the entire F protein. BALB/c mice were vaccinated with these peptides and challenged intranasally with RSV/A A2 strain. At 5 days post-challenge, mice immunized with the p27 region had significantly lower lung viral titers and pathology scores compared to mock-vaccinated mice (Lee et al., 2022), suggesting that p27 may elicit a protective immune response; however, the protective effect is unlikely due to neutralizing antibody activity. The authors instead speculate that p27 may induce antibody-dependent cell cytotoxicity (ADCC) and T cell-mediated effector functions.

Additionally, lung tissues immunostained with anti-F-p27 antisera showed p27 surface expression post-infection. A549 cells infected with RSV/A A2 showed comparable surface staining, confirming p27 surface expression *in vitro* (Lee et al., 2022). The authors attribute this to the expression of F0 on the surface of infected cells, consistent with the results seen in 2013 by Krzyzaniak et al. (2013). However, other studies have determined that F0 is inefficient in reaching or unable to reach the cell surface (Collins and Mottett, 1991; Bolt et al., 2000; Sugrue et al., 2001). A mechanistic understanding of how p27 is expressed on the cell surface has yet to be uncovered.

## 8. Gaps in knowledge and future directions

Important work in electron microscopy, crystallography, and structure modeling over the past 20 years has increased our understanding of the RSV F protein structure; however, the biological roles and fate of p27 remain elusive.

Cleavage of p27 is not a requirement for cellular transport, as the F protein can exist in a heterogenous population of uncleaved, partially cleaved, and fully cleaved F proteins on the cell surface (San-Juan-Vergara et al., 2012; Krzyzaniak et al., 2013; Lee et al., 2022). However, it is unknown if the fully cleaved p27 is secreted as free peptide or if it has an intracellular role that improves viral fitness. In addition, although the F protein sequence is well-conserved between RSV subtypes and genotypes, the p27 region is variable (Hause et al., 2017); therefore, future studies that address such sequence differences may shed light on F protein structure, entry mechanism, and infectivity (Fuentes et al., 2016).

It is unclear if complete cleavage of the F protein is required for trimerization of F1 + F2 heterodimers, although it is accepted that a partially cleaved p27 within the F protein cavity would disrupt the trimerization (Krarup et al., 2015). On the other hand, the “breathing” motion of the F protein trimer and models of prefusogenic F trimer indicate that the RSV F protein could trimerize while harboring a partially cleaved p27 (Gilman et al., 2019).

While immunological data showed that p27 elicits serum antibody responses in RSV-infected individuals of all ages, the lackluster neutralizing activity of anti-p27 IgG antibodies raises the possibility that protection might come from ADCC or other cell-mediated immune mechanisms, and this deserves further investigation (Blunck et al., 2022). Likewise, the role of mucosal anti-p27 IgA antibodies in viral clearance requires additional studies. Lastly, the humoral and mucosal immune responses to p27 might be potentially powerful biomarkers of RSV infection.

## Author contributions

WR and HN participated in the writing and preparation of the manuscript. RD and PP reviewed and approved it for publication. All authors contributed to the article and approved the submitted version.

## Funding

Discretionary funds: PP. The authors also acknowledge supplemental funding provided by Baylor Research Advocates for Student Scientists (BRASS) to WR.

## Conflict of interest

The authors declare that the research was conducted in the absence of any commercial or financial relationships that could be construed as a potential conflict of interest.

## Publisher’s note

All claims expressed in this article are solely those of the authors and do not necessarily represent those of their affiliated organizations, or those of the publisher, the editors and the reviewers. Any product that may be evaluated in this article, or claim that may be made by its manufacturer, is not guaranteed or endorsed by the publisher.
